# TPMT and NUDT15 polymorphisms in thiopurine induced leucopenia in inflammatory bowel disease: a prospective study from India

**DOI:** 10.1186/s12876-021-01900-8

**Published:** 2021-08-23

**Authors:** Narinder Grover, Prateek Bhatia, Antriksh Kumar, Minu Singh, Deepesh Lad, Harshal S. Mandavdhare, Jayanta Samanta, Kaushal K. Prasad, Usha Dutta, Vishal Sharma

**Affiliations:** Department of Internal Medicine, Pediatric Hemato-Oncology, Gastroenterology and Hematology, Postgraduate Institute of Medical Education and Research, Chandigarh, India

**Keywords:** Inflammatory bowel disease, Crohn’s disease, Ulcerative colitis, Cytopenia, Bone marrow suppression, 6-mercaptopurine

## Abstract

**Background:**

Polymorphisms in thiopurine methyltransferase (TPMT) and Nudix hydrolase-15 (NUDT15) have been implicated as the predominant cause of thiopurine induced leukopenia in the Western countries and East Asia respectively. Exact role of these polymorphisms in South Asian population with inflammatory bowel disease (IBD) is uncertain.

**Methods:**

We included consecutive patients with IBD who were initiated on thiopurines at a center in North India. The dosage of thiopurines was titrated using regular monitoring of hemogram and liver function tests. Three TPMT polymorphisms (c.238 G > C, c.460 G > A, and c.719A > G) and one NUDT15 polymorphism (c.415 C > T) were assessed. Comparison regarding incidence of leukopenia and maximum tolerated thiopurine dosage was performed between those with wild polymorphism and those with TPMT and NUDT15 polymorphisms, respectively.

**Results:**

Of the 119 patients (61 males, mean age 36.8 ± 13.5 years), 105 (88.2%) had ulcerative colitis and 14 (11.8%) had Crohn’s disease. Leukopenia was noted in 33 (27.7%), gastrointestinal intolerance in 5 (4.2%) and pancreatitis in 2 (1.6%). TPMT polymorphisms were detected amongst five patients of whom 1 developed leukopenia. NUDT15 polymorphism was noted in 13 patients of whom 7 had leukopenia. The odds of developing leukopenia in TPMT polymorphism were non-significant (0.77, 95% CI:0.0822 to 7.2134, *P* = 0.819) but were significantly higher in those with NUDT15 polymorphism (3.5933, 1.1041 to 11.6951, P value: = 0.0336).

**Conclusion:**

NUDT15 polymorphism was more frequent than TPMT polymorphisms and was associated with thiopurine induced leukopenia. However, the tested polymorphisms account for only 24.2% of the risk of thiopurine induced leukopenia.

**Supplementary Information:**

The online version contains supplementary material available at 10.1186/s12876-021-01900-8.

## Background

Thiopurine drugs are commonly used in inflammatory bowel disease (IBD) for maintenance of remission because of their immunomodulatory and steroid sparing effects [1, 2]. The availability of long-term data on the safety, efficacy and cost-effectiveness (especially in relation to biologics) make them an attractive option for use by clinicians [2, 3]. The indication for use of thiopurines in IBD include maintenance of remission in Crohn's disease (CD) and for patients with steroid dependent and refractory ulcerative colitis (UC). In recent times, the adjuvant use of thiopurines has been found to be useful to avoid formation of antidrug antibodies with anti-TNF agents [2–5]. Thiopurines act by blockade of a small GTPase of the ρ-family and inhibition of purine and protein synthesis in lymphocytes [5]. This leads to apoptosis and decreased proliferation of the T- lymphocytes and hence the immunosuppressive role in IBD.

Azathioprine is a prodrug that gets non-enzymatically converted to 6-mercaptopurine (6-MP) in the body [6]. The metabolism of 6-MP is complex and involves numerous basic intermediates, like the nucleoside triphosphates, 6-thio-GTP, 6-thio-DGTP, etc. [7]. 6-Thioguanine (6-TG) is the metabolite that is associated with not only the therapeutic effect of the drug but also its toxicity. Certain adverse effects are classified as idiosyncratic and include flue like syndrome (fever, nausea, skin rash, body ache and headache), gastrointestinal intolerance and acute pancreatitis. These are believed to be unpredictable and require drug discontinuation [8]. Cytopenias, especially leucopenia, are recognized as a dose-dependent adverse effect of thiopurines. Cytopenias have been reported with a variable frequency in patients with thiopurine use and may result in significant treatment discontinuation and occasional morbidity [4]. Various enzymes like thiopurine S-methyltransferase (TPMT), xanthine oxidase and hypoxanthine phosphoribosyl transferase are enzymes involved in the metabolism of thiopurines [9]. Myelosuppression in particular is dependent on TPMT activity which is a function of TPMT gene polymorphism and the inheritance pattern in an individual [10]. Nudix hydrolase 15 (NUDT15) hypoactivity due to certain polymorphisms, is associated with myelosuppression due to Rac1 inhibition [4, 11] The polymorphisms in the NUDT15 gene are more prevalent in East Asian population in contrast to TPMT polymorphisms which are more prevalent in the western world [11, 12]. However, the data from South Asia is limited and it is unclear which of these polymorphisms are of more importance in this setting.

Therefore, we performed a prospective study to evaluate the prevalence of TPMT and NUDT15 genetic polymorphism in the Indian patients of IBD initiated on thiopurines. We also estimated the relationship of these polymorphisms with occurrence of cytopenia in our study population.

## Methods

### Setting

The present observational cohort study was carried out in the Department of Gastroenterology, at a large tertiary care center in North India. The patients were enrolled between January 2019 to March 2020. Before conducting the study, ethical clearance was taken from the institute ethics committee. The study was done as per the ICMR guidelines**.** A written informed consent was taken from each patient included in the study.

### Patient selection and follow-up

Patients who visited outpatient’s clinic or were admitted with IBD were screened for inclusion. We included patients with inflammatory bowel disease who were started on thiopurines (azathioprine or 6-mercaptopurine). We excluded those patients who were aged ≤ 12years, refused consent to participate or were unwilling for follow-up and those with underlying conditions predisposing to bone marrow suppression like on other drugs likely to cause hematological toxicity. The analysis was done for the patients with complete follow-up of at least 3 months after initiation of the thiopurine therapy. However, the patients in whom the drug discontinuation was warranted for an adverse effect were included in the analysis irrespective of the timing of this event.

The details of all included patients regarding demographic and clinical details, underlying diagnosis, Montreal classification of IBD, were recorded in a predesigned case record form. The details regarding the clinical presentation, age of onset of symptoms, details of disease extent and duration were recorded. The treatment received was purely guided by the patient’s medical condition and discretion of the treating physician. The starting dose of azathioprine was 1 mg/kg and for 6 mercaptopurine was 0.5 mg/kg. After checking the hemogram at 2 weeks, if there was no leukopenia, we increased the dose of azathioprine by 0.5 mg/kg or 6-mercaptopurine by 0.25 mg/kg to reach a maximal dose of 2.5 mg/kg or 1 mg/kg respectively. After any increase in the dosage of thiopurine analogues, the patient was followed up at two weeks with complete blood count (CBC). Leukopenia was defined as a total leucocyte count < 3000/cumm. The details of the thiopurine therapy i.e., agent used, initial dose, maximum tolerated dose (in azathioprine equivalents), any adverse effects including leukopenia, gastrointestinal intolerance, hepatitis or pancreatitis were recorded.

### Assessment of genetic polymorphism

Two–three ml of blood sample was withdrawn from individuals via venipuncture and was collected in EDTA vial. The blood sample was used in testing for thiopurine analogues metabolism related polymorphism (TPMT and NUDT15). The blood sample taken in EDTA tubes was assigned.

a unique accession number and DNA from 200 µl of blood was extracted. This was done using QI Amp DNA blood kit (Qiagen Inc.). DNA was eluted in 50 µl of elution buffer and was kept at − 20 °C until analysis.

### Genotyping

Three sites of known TPMT gene mutations causing TPMT deficiency (c.238 G > C, c.460 G > A, and c.719A > G) were determined according to the method described by Yates et al. [13]. Briefly, Amplification Refractory Mutation System (ARMS) PCR was used to detect the c.238 G > C transversion in exon 5, while PCR amplification and restriction enzyme digestion (PCR–RFLP) to detect the c.460 G > A and c.719 A > G mutations in exon 7 and 10 using enzymes MwoI and AccI, respectively. To detect c.415 C > T mutation in NUDT15, PCR–RFLP method was employed as described previously [14].

### Sample size calculation

Keeping the possibility that 20% of patients on thiopurine analogues would develop leukopenia and expecting that 50% of them to have some detectable mutation as compared to around 10% in those who do not develop any leukopenia, the calculated sample size was 78 with power of 80% and 2-sided confidence level of 95%. Keeping 20% loss to follow up in mind we planned to enroll 100 patients.

### Comparison and data analysis

At the end of the study, side effects such as leukopenia and idiosyncratic reactions were compared between patients having TPMT and NUDT15 polymorphism and patients with no detectable polymorphisms. For analysis, we grouped those with flue like syndrome (fever, nausea, skin rash, body ache and headache), gastrointestinal intolerance and acute pancreatitis into a single group i.e. idiosyncratic. The categorical data were compared using Chi square test. For continuous variables the normality of data was checked using Kolmogorov–Smirnov test and appropriate comparison (using Student’s T test or Mann Whitney test) was done. We also compared the patients who developed leukopenia and those who did not develop leukopenia for baseline characteristics (age, gender, basic diagnosis, use of 5-aminosalicylates, recent steroid use, baseline values of hemogram, leucocyte count and platelets) to identify any predictors of development of leukopenia. Multivariate analysis was performed to identify independent predictors of leukopenia.

## Results

### Patients

**Fig. 1:**
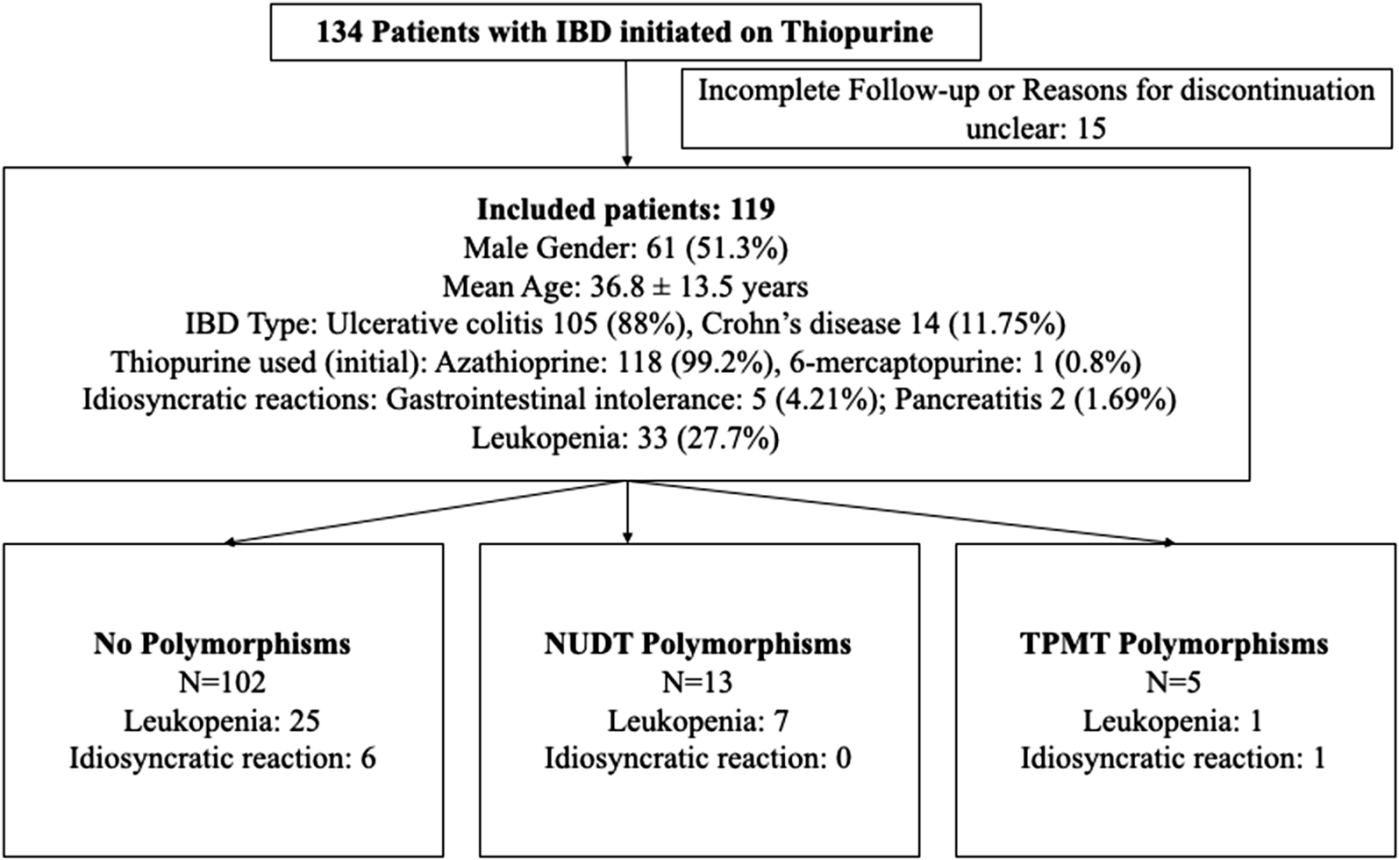
The flow of patient recruitment and evaluation

A total of 134 patients were initiated on thiopurine therapy during the study period. Fifteen patients were lost to follow up or did not complete 3 months of follow-up on thiopurines. One hundred and nineteen patients were included in the final analysis amongst which 61 (51.3%) were males. The mean age was 36.8 ± 13.5 years. Of 119 patients, 105 had ulcerative colitis while 14 had Crohn’s disease. Of those UC patients in whom the extent was evaluated (n = 74), majority had extensive disease. Of the 14 patients with CD, 7 had ileocolonic disease while rest of 7 had ileal disease. Overall steroid and 5-amino salicylate use were high in our population because most were treated for active disease (54 had recent acute severe ulcerative colitis and 12 had active Crohn’s disease). Five patients on azathioprine were shifted to 6-MP because of gastrointestinal intolerance (4 cases) and pancreatitis (1 patient) which occurred with azathioprine. Leukopenia were noted amongst 33 (27.7%), gastrointestinal tolerance in 5 (4.2%) and pancreatitis in 2 (1.6%) patients. None of the patients developed liver injury. Early leukopenia (within 8 weeks) was noted in six patients while 27 developed late leukopenia. Treatment interruptions (stopping or decreasing the dosage) were required in 37 (31.1%) patients. TPMT polymorphisms were noted amongst five patients while NUDT15 polymorphism was noted in 13 patients. One of them had both TPMT and NUDT15 polymorphism (Fig. 1).

### TPMT polymorphism

The parameters between the two groups (TPMT polymorphism present or no polymorphism) were similar for the baseline hemoglobin, leucocyte and platelet counts (Table 1). One out of the five patients who had TPMT polymorphism developed leukopenia and one developed an idiosyncratic reaction whereas 25 of the 102 patients in no polymorphism group developed leukopenia and six developed idiosyncratic reactions (Table 2). The differences were not statistically different and the odds of developing leukopenia in TPMT group were 0.77 (95% CI:0.0822 to 7.2134, *P* = 0.819). The maximum tolerated dose was similar between the two groups (80 ± 37.1and 91.4 ± 28.0 mg/day).

**Table 1:** Comparison of baseline characteristics amongst patients with TPMT, with NUDT15 and without any mutations

	TPMT mutated (N = 5) n (%)/ Median (IQR)	Wild Genotype (N = 102) n (%)/ Median (IQR)	*P* value	NUDT15 mutated (N = 13) n (%)/ Median (IQR)	Wild Genotype (N = 102) n (%)/ Median (IQR)	*P* value
Age (years)	42 (16)	35.5 (22)	0.881	40 (19)	35.5 (22)	0.418
Male	1 (20%)	53 (51.9%)	0.162	7 (53.8%)	53 (51.9%)	0.898
Smoker (past or present)	0	16 (15.7%)	1.00	2 (15.4%)	16 (15.7%)	1.000
Alcohol use (past or present)	0 (100%)	24 (23.5%)	1.00	2 (15.4%)	24 (23.5%)	0.729
Body weight (kg)	55 (18)	55 (20)	0.915	54 (8)	55 (20)	0.407
Comorbidities	0 (0%)	14 (13.7%)	1.00	3 (23.1%)	14 (13.7%)	0.405
Recent Steroid Use (3 months)	2 (40%)	58 (56.9%)	0.652	7 (53.8%)	58 (56.9%)	1.000
Current 5-Aminosalisylate use	5 (100%)	97 (95.1)	1.000	12 (92.3%)	97 (95.1)	0.5214
Disease duration (months)	48.0 (138)	36 (41)	0.949	36 (62)	36 (41)	0.168
Hemoglobin (g/dL)	10.7 (4.81)	11.2 (2.93)	0.949	12.8 (2.35)	11.2 (2.93)	0.236
Total leucocyte count (× 10^6^/L)	9700 (4080)	8950 (4400)	0.348	9700 (4750)	8950 (4400)	0.477
Platelet count (× 10^6^/L)	354,000 (104,000)	323,500 (183,250)	0.327	292,000 (186,000)	323,500 (183,250)	0.516

**Table 2:** Outcomes of thiopurine therapy in patients with or without TPMT polymorphisms

	TPMT mutated (N = 5) n (%)/ Median (IQR)	Wild genotype (N = 102) n (%)/ Median (IQR)	*p* value
Hemoglobin (g/dL) at the end of follow up	10.8 (3.7)	11.05 (3.4)	0.983
Total leucocyte count (× 10^6^/L) at the end of follow up	6340 (4200)	5700 (3575)	0.949
Platelet count (× 10^6^/L) at end of follow up	312,000 (161,000)	254,000 (132,500)	0.983
Duration of thiopurine use (months)	12 (14.9)	8 (15.25)	0.348
Max tolerated azathioprine equivalent dose (mg/d)	75 (50)	100 (25)	0.448
Leukopenia	1 (20%)	25 (24.5%)	1.000
Idiosyncratic reaction	1 (20%)	6 (5.9%)	0.292
Treatment interruption	1 (20%)	29 (28.4%)	0.532

### NUDT15 polymorphism

The baseline parameters were similar between the group with or without NUDT15 polymorphism (Table 1). Of the 13 patients with NUDT15 polymorphism, 7 developed leukopenia in comparison to 25 in the non-polymorphism group of whom two had hair fall (Table 3). The odds of developing leukopenia in the NUDT15 polymorphism group were 3.5933 (95% CI, 1.1041 to 11.6951, *P* value: = 0.0336). The median TLC at the treatment commencement was similar in the mutated and non-mutated patients, however, on follow up, a significant drop was observed in the patients with NUDT15 mutation (*p* = 0.006). None of the patients in the NUDT15 group developed any idiosyncratic reaction.

**Table 3:** Outcomes of thiopurine therapy in patients with or without NUDT15 polymorphisms

	NUDT15 (mutated) (N = 13) n (%)/ Median (IQR)	Wild Genotype (N = 102) n (%)/ Median (IQR)	*p* value
Hemoglobin (g/dL) at end of follow up	12 (2.8)	11.05 (3.4)	0.289
Total leucocyte count (× 10^6^/L) at end of follow up	3600 (1700)	5700 (3575)	**0.006**
Platelet count (× 10^6^/L) at end of follow up	214,000 (109,000)	254,000 (132,500)	0.289
Duration of thiopurine use (months)	11 (9)	8 (15.25)	0.168
Max tolerated azathioprine equivalent dose (mg/d)	50 (50)	100 (25)	0.253
Leukopenia	7 (53.8%)	25 (24.5%)	**0.026**
Idiosyncratic reaction	0	6 (5.9%)	1.000
Treatment interruption	7 (53.8%)	29 (28.4%)	0.063

Bold means statistically significant

### Predictors of leukopenia

On univariate comparison of patients developing leukopenia and those who did not, significant differences were observed for the age and presence of NUDT15 polymorphisms (Additional file 1: Table S1). For binary logistic regression all parameters which attained a significance at *P* < 0.1 were entered in the model (age, disease duration, duration of use of thiopurine, total leucocyte count at start, alcohol use and presence of NUDT15 polymorphism). Eventually, total leucocyte count, and presence of NUDT15 polymorphism were found to be independent predictors of leukopenia (Additional file 1: Table S2). The odds for prediction of leukopenia were exactly 1.00 for baseline TLC but were 5.229 (1.437–19.035) for presence of NUDT15 polymorphism.

## Discussion

In this study, leukopenia was noted in 27.7% of patients with inflammatory bowel disease who were initiated on thiopurine treatment. The frequency of idiosyncratic reaction was much lower (5.9%). The frequency of NUDT15 polymorphism was higher than TPMT polymorphisms in Indian patients with IBD (10.9% versus 4.2%). The NUDT15 polymorphism correlated with occurrence of leukopenia (53.8% of those with NUDT15 polymorphism). The presence of TPMT polymorphisms did not correlate with the occurrence of leukopenia nor did it affect the maximal tolerated azathioprine equivalent dose. On multivariate analysis also, presence of NUDT15 polymorphism was independent predictor of development of leucopenia.

Thiopurines, an important armamentarium in the management of IBD, have certain adverse effects. Occurrence of leukopenia is an important concern associated with these drugs and is associated with significant interruptions in the treatment [1, 2]. In recent years, polymorphisms in the genes for enzymes involved in thiopurine metabolism have been recognized to be predict occurrence of cytopenia. These developments have brought the field personalized medicine into clinical use. However, the important polymorphisms responsible for causing leukopenia vary amongst different populations. In Western countries, polymorphisms in TPMT gene have been recognized as the important cause of thiopurine related leukopenia [15–17]. However, polymorphisms in NUDT15 are believed to be more important contributor to occurrence of leukopenia in East Asia [4, 18].

Our findings from the present study suggest that NUDT15 polymorphism is more prevalent than TPMT polymorphisms in Indian patients and should be tested before initiating thiopurine therapy in Indian population. Our findings support the use of NUDT15 testing for individualizing dose of thiopurine in patients with IBD. The data in the South Asian population about the role of TPMT and NUDT15 polymorphisms is limited. In a study from Western India in 69 patients with IBD, NUDT15 polymorphism was present in 9 (13%) whereas TPMT polymorphisms were not detected [19]. Further, these findings also suggest the need to look for additional polymorphisms/ mutations because a substantial number of patients (around 25%) with thiopurine induced leukopenia did not have identifiable polymorphisms amongst those which were tested. Another recent study from India including more than 1000 patients noted leucopenia in only 9% and this was associated with detectable mutation in 59 patients (54 had NUDT15 polymorphism) [20]. Interestingly, in contrast our study had a much higher leucopenia and also known polymorphisms accounted only for a quarter of the leucopenia. These differences could be related to certain key differences in our study including prospective follow-up, inclusion of more patients with active disease and protocol-based increments in thiopurine dosages resulting in higher mean dose used. Our findings are consistent with a recent systematic review on prevalence of polymorphisms in thiopurine in South Asian region which suggested similar higher prevalence of NUDT15 polymorphism in healthy controls and non-IBD diseased populations also [21]. In consonance with recent reports of severe and fatal toxicity of thiopurine in those homozygous for NUDT15 polymorphisms, our study makes a strong case for pre-emptive testing for these in South Asian populations [22, 23]. Further, in centers and populations where the biologic use is not common including those where steroids and cyclosporine (as second line therapy in acute severe colitis) continues to be used for IBD, azathioprine is an important drug for maintenance of remission and therefore preemptive testing of NUDT15 polymorphisms may help improve tolerance to thiopurine therapy [21, 24].

The present study has some limitations: the number of patients with CD were much less than the number of UC. Further, we did not measure the levels of 6-thioguanine to correlate with the cytotoxicity and also that the study was done at a single center in North India. However, the prospective nature of the study, testing of both TPMT and NUDT15 polymorphisms and adequate duration of follow-up are the strengths of this study. The study provides information which would be important not just for the Indian population but also to Western countries with significant migrant populations from South Asia.

## Conclusion

The present study suggests that in South Asian populations with IBD the polymorphisms in NUDT15 gene may have a more important role in determining the occurrence of thiopurine related cytopenia than TPMT. Further, the four polymorphisms tested only explain 21% of the overall risk of leukopenia suggesting the need to look for novel mutations.

## Supplementary Information

**Additional file 1**.** Table S1**: Univariate analysis to identify the predictors of cytopenia.** Table S2**: Multivariate analysis of predictors for cytopenia

## Data Availability

The data can be provided by the corresponding author on reasonable request.

## Abbreviations

TPMT: Thiopurine methyltransferase
NUDt-15: Nudix hydrolase-15
UC: Ulcerative colitis
CD: Crohn’s disease
IBD: Inflammatory bowel disease
anti-TNF: Anti-tumour necrosis factor
GTP: Guanosine triphosphate
DGTP: Deoxyguanosine triphosphate
6-MP: 6-Mercaptopurine
ICMR: Indian Council of Medical Research
ARMS: Amplification Refractory Mutation System
PCR: Polymerase chain reaction
RFLP: Restriction enzyme digestion

## References

[1] Saibeni S, Virgilio T, D'Incà R, Spina L, Bortoli A, Paccagnella M (2008). The use of thiopurines for the treatment of inflammatory bowel diseases in clinical practice. Dig Liver Dis.

[2] Fraser AG, Orchard TR, Jewell DP (2002). The efficacy of azathioprine for the treatment of inflammatory bowel disease: a 30 year review. Gut.

[3] Vasudevan A, Parthasarathy N, Con D, Nicolaides S, Apostolov R (2020). Thiopurines vs methotrexate: comparing tolerability and discontinuation rates in the treatment of inflammatory bowel disease. Aliment Pharmacol Ther.

[4] Matsuoka K (2020). NUDT15 gene variants and thiopurine-induced leukopenia in patients with inflammatory bowel disease. Intest Res.

[5] Seinen ML, van Nieuw Amerongen GP, de Boer NK, van Bodegraven AA (2016). Rac attack: modulation of the small GTPase rac in inflammatory bowel disease and thiopurine therapy. Mol Diagn Ther.

[6] Elion GB (1989). The purine path to chemotherapy. Science.

[7] van Gennep S, Konté K, Meijer B, Heymans MW, D'Haens GR, Löwenberg M, de Boer NKH (2019). Systematic review with meta-analysis: risk factors for thiopurine-induced leukopenia in IBD. Aliment Pharmacol Ther.

[8] van Geenen EJ, de Boer NK, Stassen P (2010). Azathioprine or mercaptopurine-induced acute pancreatitis is not a disease-specific phenomenon. Aliment Pharmacol Ther.

[9] Van Scoik KG, Johnson CA, Porter WR (1985). The pharmacology and metabolism of the thiopurine drugs 6-mercaptopurine and azathioprine. Drug Metab Rev.

[10] Nguyen CM, Mendes MA, Ma JD. Thiopurine methyltransferase (TPMT) genotyping to predict myelosuppression risk. *PLoS Curr*. 2011;3:RRN1236. 10.1371/currents.RRN1236 PMC3094768 21593964

[11] Singh M, Bhatia P, Khera S, Trehan A (2017). Emerging role of NUDT15 polymorphisms in 6-mercaptopurine metabolism and dose related toxicity in acute lymphoblastic leukaemia. Leuk Res.

[12] Kakuta Y, Kinouchi Y, Shimosegawa T (2018). Pharmacogenetics of thiopurines for inflammatory bowel disease in East Asia: prospects for clinical application of NUDT15 genotyping. J Gastroenterol.

[13] Yates CR, Krynetski EY, Loennechen T (1997). Molecular diagnosis of thiopurine S-methyltransferase deficiency: genetic basis for azathioprine and mercaptopurine intolerance. Ann Intern Med.

[14] Khera S, Trehan A, Bhatia P, Singh M, Bansal D, Varma N (2019). Prevalence of TPMT, ITPA & NUDT 15 genetic polymorphisms and their relation to 6MP toxicity in north Indian children with Acute Lymphoblastic Leukemia. Cancer Chemother Pharmacol.

[15] Walker GJ, Harrison JW, Heap GA, Voskuil MD, Andersen V, Anderson CA, et al; IBD Pharmacogenetics Study Group. Association of Genetic Variants in NUDT15 With Thiopurine-Induced Myelosuppression in Patients With Inflammatory Bowel Disease. JAMA. 2019;321(8):773–85. 10.1001/jama.2019.0709 PMC6439872 30806694

[16] Carvalho ATP, Esberard BC, Fróes RSB, Rapozo DC, Grinman AB, Simão TA (2014). Thiopurine-methyltransferase variants in inflammatory bowel disease: prevalence and toxicity in Brazilian patients. World J Gastroenterol.

[17] Reuther LO, Sonne J, Larsen N, Dahlerup JF, Thomsen OO, Schmiegelow K (2003). Thiopurine methyltransferase genotype distribution in patients with Crohn’s disease. Aliment Pharmacol Ther.

[18] Chang JY, Park SJ, Jung ES, Jung SA, Moon CM, Chun J, Park JJ, Kim ES, Park Y, Kim TI, Kim WH, Cheon JH (2020). Genotype-based Treatment With Thiopurine Reduces Incidence of Myelosuppression in Patients With Inflammatory Bowel Diseases. Clin Gastroenterol Hepatol.

[19] Shah SA, Paradkar M, Desai D, Ashavaid TF (2017). Nucleoside diphosphate-linked moiety X-type motif 15 C415T variant as a predictor for thiopurine-induced toxicity in Indian patients. J Gastroenterol Hepatol.

[20] Banerjee R, Ravikanth VV, Pal P, Bale G, Avanthi US, Goren I, Girish BG, Mitnala S, Reddy DN (2020). NUDT15 C415T variant compared with TPMT genotyping in predicting azathioprine-induced leucopenia: prospective analysis of 1014 inflammatory bowel disease patients in India. Aliment Pharmacol Ther.

[21] Jena A, Jha DK, Kumar-M P, Kasudhan KS, Kumar A, Sarwal D, Mishra S, Singh AK, Bhatia P, Patil A, Sharma V (2021). Prevalence of polymorphisms in thiopurine metabolism and association with adverse outcomes: a South Asian region-specific systematic review and meta-analysis. Expert Rev Clin Pharmacol.

[22] Yadav A, Jena A, Thakur R, Kumar P, Bhatia P, Sharma V (2021). Fatal thiopurine toxicity: pre-emptive testing of *NUDT15 415C*>*T* polymorphism may be life saving in South Asian population. Drug Metab Pers Ther.

[23] Devasia AJ, Illangeswaran RSS, Raj IX, George B, Balasubramanian P (2020). NUDT15 polymorphism explains serious toxicity to azathioprine in Indian patients with chronic immune thrombocytopenia and autoimmune hemolytic anemia: a case series. Drug Metab Pers Ther.

[24] Mishra S, Mandavdhare HS, Singh H, Choudhury A, Shah J, Ram S, Kalsi D, Samanta J, Prasad KK, Sharma AK, Dutta U, Sharma V (2020). Adjuvant use of combination of antibiotics in acute severe ulcerative colitis: A placebo controlled randomized trial. Expert Rev Anti Infect Ther.

