# Supplementary material for: TPMT and NUDT15 polymorphisms in thiopurine induced leucopenia in inflammatory bowel disease: a prospective study from India
Source: BMC Gastroenterol. 2021 Aug 23;21:327. doi: 10.1186/s12876-021-01900-8 (PMC8383411; doi:10.1186/s12876-021-01900-8)
Supplement: Supplementary file 1 — Additional file 1. Table S1: Univariate analysis to identify the predictors of cytopenia. Table S2: Multivariate analysis of predictors for cytopenia [file 12876_2021_1900_MOESM1_ESM.docx]

Supplementary Table 1: Univariate analysis to identify the predictors of cytopenia

|  | **Cytopenia (N=33)**  **N(%)/median (IQR)** | **No Cytopenia (N=86)**  **N(%)/median (IQR)** | **p-Value** |
| --- | --- | --- | --- |
| Male gender (n=61) | 19 (57.6%) | 42 (48.8%) | 0.393 |
| Smoking (n=18) | 7 (21.2%) | 11 (12.8%) | 0.251 |
| Alcohol use(n=26) | 11 (33.3%) | 15 (17.4%) | *0.060* |
| Subtype  UC (n=105)  CD (n=14) | 27 (81.8%)  6 (18.1%) | 78 (90.7%)  8 (9.3) | 0.178 |
| TPMT Polymorphism | 1 (3.03%) | 4 (4.7%) | 0.693 |
| NUDT15 polymorphism | 7 (21.2%) | 6 (6.9%) | **0.026** |
| Age (years) | 44 (18) | 34.5 (20) | **0.011** |
| Disease duration (months) | 48 (48) | 33 (43) | **0.047** |
| Body weight (kg) | 55 (16) | 55 (19) | 0.535 |
| Duration of drug use (months) | 11 (19.5) | 8 (9) | **0.011** |
| Hb (gram/dL) at treatment initiation | 11.3 (2.35) | 11.3 (3.25) | 0.563 |
| TLC (per mm^3^ ) at treatment initiation | 8540 (5150) | 9600 (5050) | 0.067 |
| Platelet at treatment initiation | 316000(139000) | 326000(196750) | 0.358 |

UC: Ulcerative colitis; CD: Crohn’s disease; TPMT: thiopurine methyltransferase; NUDT15: Nudix hydrolase-15 ; Hb: Hemoglobin; TLC: total leucocyte count

Supplementary Table 2: Multivariate analysis of predictors for cytopenia

|  | Odds ratio (95% CI) | p-value |
| --- | --- | --- |
| Age | 1.021(0.985-1.058) | 0.251 |
| Alcohol use | 2.391 (0.829-6.891) | 0.107 |
| Duration of drug use | 1.018 (0.996-1.042) | 0.112 |
| TLC at treatment initiation | 1.000(1.000-1.000) | **0.025** |
| NUDT15 polymorphism | 5.229(1.437-19.035) | **0.012** |
| Duration of disease | 1.001(0.992-1.011) | 0.831 |

NUDT15: Nudix hydrolase-15 ; TLC: total leucocyte count
